# Interventions to Address Racism in Disciplinary Actions in K-12 Schools: A Systematic Review

**DOI:** 10.1007/s11121-025-01855-2

**Published:** 2025-11-22

**Authors:** Briana A. Scott, Sarah M. Stilwell, Zaida V. Pearson, Marc A. Zimmerman, Hsing-Fang Hsieh, Justin Heinze

**Affiliations:** Institute for Firearm Injury Prevention, University of Michigan, Ann Arbor, MI USA

**Keywords:** School intervention, Racism, School safety, Discipline gap, School discipline

## Abstract

Students of color are disciplined for behavioral infractions at higher rates than white students in K-12 schools in the USA. The consequences of racism in K-12 schools include mental health problems, school dropout, and disproportionate disciplinary practices, leading to the school-to-prison pipeline. Many school personnel implement interventions to address student behavior rather than racism and other implicit biases. Furthermore, culturally relevant practices are imperative to address the root causes of racial disparities in student discipline. For these reasons, a systematic and comprehensive review of the published literature on school-based interventions in the USA (including public and private K-12 schools) was conducted to identify interventions to remedy racial disparities in school discipline, as well as the research designs used to test their efficacy. The final sample includes 48 studies that directly or indirectly attempt to address the race discipline gap. There were only three studies that reduced race disparities in school discipline with a culturally relevant intervention. Future researchers may consider the importance of the school’s cultural context and intervention audience when developing and testing efforts to reduce racial disproportionality.

Racial disparities in school disciplinary practices remain a pervasive challenge in K-12 education in the USA (Rausch & Skiba, 2017; Welsh & Little, 2018). Disproportionate use of exclusionary discipline, such as suspensions and expulsions, significantly and negatively affects students of color, contributing to unequal academic, social, and long-term life outcomes, including the reinforcement of the school-to-prison pipeline (Balfanz et al., 2015; Novak, 2018; Rosenbaum, 2020). While schools have implemented a range of interventions to address disciplinary inequities, many efforts fall short by overlooking the central role of race and cultural context (Carter et al., 2017; Gregory et al., 2021; Skiba et al., 2022). This review systematically examines interventions specifically designed to address racism in disciplinary practices, with particular attention to the importance of culturally relevant approaches in mitigating the race discipline gap and promoting educational equity (Gregory et al., 2014; Ladson-Billings, 1995; Skiba et al., 2022).

## Disproportionate Discipline and Outcomes for Students

Exclusionary discipline includes a range of punitive practices that remove students from their learning environment for behavioral reasons (Zinsser et al., 2022). For instance, an office discipline referral may be issued for subjective behaviors like “defiance” or “disrespect,” or for more clearly defined incidents such as fighting. Other exclusionary practices include in-school suspension, where a student is separated from peers for a period during the school day; out-of-school suspension, which entirely removes a student from school premises for one or more days; expulsion, which can exclude a student for the remainder of a term or school year; alternative school placements; and referral to the juvenile justice system (Gerlinger et al., 2021; Zinsser et al., 2022). Common student behaviors that result in exclusionary discipline fall into different categories of infractions. More severe actions, such as physical aggression, may be addressed differently from less severe behaviors like disobedience or insubordination. Other frequently cited reasons for exclusionary discipline include chronic absenteeism and poor academic performance (Balfanz et al., 2015; Zinsser et al., 2022). Exclusionary discipline is common in schools, with approximately 2.4 million students receiving one or more out-of-school suspensions during the 2021–22 school year, and over 28,500 were expelled (U.S. Department of Education, 2025). Exclusionary discipline is associated with the school-to-prison pipeline, which can be understood as the policies and practices that directly or indirectly push students out of school and on a trajectory to prison (Novak, 2018).

Despite the ubiquity of school discipline, researchers have demonstrated that exclusionary discipline results in many adverse outcomes for students, including lost instructional time (Losen & Whitaker, 2017), lowered academic outcomes (Noltemeyer et al., 2015), and increased likelihood of truancy and dropout (Balfanz et al., 2015; Rumberger & Losen, 2017). Furthermore, experiencing suspension or expulsion during adolescence increases the chances of involvement in the criminal justice system and results in lower educational attainment during adulthood (Rosenbaum, 2020; Wolf & Kupchik, 2017). Additionally, higher suspension and expulsion rates have been associated with reduced schoolwide academic performance (Skiba & Rausch, 2006) and perceptions of school climate (Gregory et al., 2011). Taken together, a robust body of empirical evidence demonstrates that exclusionary discipline has negative implications for students throughout their development, and even more so for students of color.

### Causes of Disproportionate Discipline

For the purposes of this study, racism is defined as both individual and systemic actions, practices, and ideologies that produce and maintain racial inequities in disciplinary outcomes, whether actively or through implicit bias and institutional norms (Skiba et al., 2014). In the school discipline context, this includes policies and practices that disproportionately and unjustly impact students of color, regardless of intent. While racial disparities refer to measurable differences in discipline rates across groups, racism is understood as the underlying cause of these disparities. Theory and research demonstrate that disciplinary gaps persist even when the student behaviors remain the same and are instead driven by biased decision-making, discretionary policies, and broader institutional structures (Gregory et al., 2011; Skiba et al., 2011). Thus, this paper frames discipline disparities not as isolated outcomes, but as manifestations of racism embedded within educational systems.

Significant racial disparities in school discipline have been reported in both highly resourced and under-resourced school districts (Skiba & Rausch, 2014), in both public and charter schools (Losen et al., 2016), and at the elementary, middle, and high school levels (Losen et al., 2015). Racial disproportionality in school discipline is observed across multiple racial minority groups (Cruz et al., 2021). For example, Black students are more likely to receive an office discipline referral (ODR) compared to their White counterparts (Martinez et al., 2016). Moreover, the ODRs that Black students receive are often for subjective infractions (e.g., those that require teacher judgment) rather than objective or behavioral (e.g., truancy, physical aggression), compared to the ODRs that White students receive, leaving more room for bias (Smolkowski et al., 2016). When a student is harshly disciplined in school, it can lead to them being officially labeled by school personnel as problematic or dangerous (Kennedy-Lewis & Murphy, 2016). The consequences of this label for a student of color are more severe because this action fulfills a stereotype many people have about students of color as dangerous and disobedient (Okonofua et al., 2016). Any future student misbehavior and discipline referrals only build the evidence among school staff that the student is a problem, and they may begin to treat the student as such. Consequently, the student may start to withdraw from school engagement and eventually drop out of school, which may result in a self-fulfilling prophecy and put them at a high risk for future unemployment and further increase the risk of future criminal behavior (Bleakley & Bleakley, 2018; Owens et al., 2016). This has ramifications for youth both in the immediate and long term, making this a crucial issue to address to ensure positive life outcomes.

Researchers have reported that exclusionary discipline disproportionately affects students of color (Rausch & Skiba, 2017; Welsh & Little, 2018), and researchers across multiple contexts and communities have well documented this disparity. Skiba and colleagues 2016 demonstrate that Black and Hispanic students are at a greater risk for suspension or expulsion for the same behavior as their White peers across all grade levels. While Black boys represent 8% of K-12 student enrollment in the USA, 22% have received one or more out-of-school suspensions, and 21% have been expelled (U.S. Department of Education, 2025). In contrast, White boys represent 23% of K-12 student enrollment, and 24% have been expelled (U.S. Department of Education, 2025).

During the 2011–12 school year, Losen and colleagues 2015 found that 16% of all Black students had been suspended, a rate that is more than double that of Hispanic students (7%) and triple the rate for White students (5%) across the USA. Furthermore, researchers found that Hispanic students are more likely to receive a suspension compared to their White peers for comparable behavior infractions in elementary (Skiba et al., 2011) and high school (Finn & Servoss, 2015) levels. These disparities represent an increasingly urgent issue given the discrepancies in the way students of color are being disciplined at a higher rate than others, and these disproportionate rates of exclusionary discipline contribute to a race-based gap that is critical to understand from an evidence-based perspective (Anderson et al., 2019).

### Previous Attempts to Curb Disproportionate Discipline

School leaders may address this inequity by incorporating race-conscious, culturally relevant school programming. Examples of this may include providing school programs in students’ preferred languages and employing educators and other school staff with shared identities of the students (Ladson-Billings, 1995). Culturally relevant school programs are designed to uplift, value, and encourage students’ diverse identities, cultural practices, traditions, and life experiences (Ladson-Billings, 1995; Vélez-Agosto et al., 2017). School discipline can negatively affect all students (Skiba et al., 2022), and researchers have found that even students who are not directly disciplined still experience negative collateral consequences affecting their health and overall well-being (Eyllon et al., 2022). Students who behave according to school rules may become preoccupied with avoiding discipline in a highly punitive environment, leading to anxiety and distress (Eyllon et al., 2022). Additionally, students who attend highly punitive schools may perceive the school environment as hostile, leading to disengagement or shame about their school, even if they are not disciplined directly (Eyllon et al., 2022). Furthermore, students of color experience the most severe ramifications of exclusionary discipline, affecting their academic performance, school connectedness, and well-being (Gregory et al., 2014), pointing to the need for efforts to address this problem.

Attention regarding how a student’s racial identity affects their experiences with the school disciplinary system is necessary to understand and address disparities (Edwards, 2021; Gregory et al., 2021). Despite numerous researchers demonstrating that Black students are disciplined at a higher rate than their non-Black peers for similar infractions (Ksinan et al., 2019; Skiba et al., 2022), interventions tend to be designed to focus on making disciplinary actions more equitable for all students, leaving out discussions of racism as a source of this disparity (Gregory et al., 2021; Skiba et al., 2022).

School leaders have developed and widely adopted school discipline practices to address the race discipline gap. Examples of these practices include Schoolwide Positive Behavioral Interventions and Supports (SWPBIS), Social-Emotional Learning (SEL), and Restorative Practices (Skiba et al., 2022). SWPBIS is a schoolwide framework that establishes and reinforces clear behavioral expectations to reduce disciplinary incidents and improve school climate (Noltemeyer et al., 2019). SEL programs focus on teaching students skills in self-management, relationship-building, and responsible decision-making to foster emotional and social well-being (Jones & Doolittle, 2017). Restorative Practices aim to build community and address the root causes of misbehavior by focusing on repairing harm and restoring relationships, rather than using punitive measures alone (Skiba et al., 2022).

While researchers have reported that these practices can reduce school disciplinary actions by improving the school climate, racial inequities persist (Sobti & Welsh, 2023). For example, researchers evaluating the efficacy of SWPBIS, a program designed to recognize and enforce student behavioral expectations consistently, found significant reductions in suspensions and ODRs for students overall (Noltemeyer et al., 2019). Other researchers, however, have found inconclusive results on racial disparities in disciplinary practices in their evaluations of SWPBIS (Gregory et al., 2021). When SWPBIS does not include a focus on race, equity, and cultural relevance, it may do little to reduce racialized discipline practices and result in continuing to harm students of color differentially (Vincent et al., 2015). Although school leaders develop and implement interventions with positive intentions, interventions designed to reduce the race discipline gap tend to take a colorblind approach, or remove race from the conversation, about the causes and solutions to disparities in discipline rates (Carter et al., 2017). Disparities in disciplinary outcomes are rooted in racism, as seen with the school-to-prison pipeline (Skiba et al., 2014). Thus, interventions that continue to take a colorblind approach are unlikely to mitigate the race discipline gap (Sobti & Welsh, 2023).

Furthermore, higher racialized disparities are found in schools with administrators who endorse zero-tolerance approaches to discipline (Huang & Cornell, 2021). Schools with overly punitive or zero-tolerance policies often rely on school resource officers or other law enforcement to respond to student misbehavior, contributing to the pipeline (Turner & Beneke, 2020). The presence of school resource officers increases exclusionary discipline for Black and Hispanic students disproportionately to that of White students (Crosse et al., 2022). In addition, the arrest rate increases for all students in schools with school resource officers, but Black students are more likely than White or Hispanic students to be arrested (Fisher & Hennessy, 2016).

### Importance of Culturally Relevant Practices

Asset-based pedagogies, including culturally relevant, culturally responsive, and culturally sustaining teaching, provide the foundation for interventions designed to promote equity in school discipline. Culturally relevant pedagogy, developed by Ladson-Billings (1995), promotes academic success, cultural competence, and critical consciousness, especially for students from marginalized backgrounds. Culturally responsive teaching emphasizes connecting instruction and relationships to students’ cultural assets and experiences (Hammond, 2015), while culturally sustaining pedagogy supports and maintains students’ cultural and linguistic practices as vital components of schooling (Paris & Alim, 2017).

These frameworks move beyond colorblind or neutral strategies by validating students’ identities and lived experiences and intentionally integrating their cultures into all aspects of schooling, including curriculum, instruction, relationships, policies, and disciplinary practices (Ladson-Billings, 1995; Vélez-Agosto et al., 2017). Culturally responsive practices are associated with higher academic achievement, improved student engagement, and a stronger sense of belonging (Vélez-Agosto et al., 2017). In discipline, such practices promote equitable school climates by challenging bias and ensuring that disciplinary policies and interactions are just and contextually informed (Gregory et al., 2014).

The present study applies a race-conscious lens to examine how interventions use asset-based pedagogies to address the root causes of racial discipline disparities. Centering these frameworks highlights the potential for interventions that view students’ backgrounds as strengths while also addressing the institutional racism that perpetuates exclusionary discipline patterns (Edwards, 2021; Skiba et al., 2022). Interventions described as culturally relevant, responsive, or sustaining are emphasized, as these approaches hold substantial promise for fostering equitable disciplinary practices.

### Current Study

Racial disparities exist in school discipline, and a plethora of evidence demonstrates this problem. To begin understanding the state of evidence, a few systematic reviews have been conducted to address this issue. For example, Cruz and colleagues (2021) conducted a best-evidence synthesis, an approach that identifies studies using explicit criteria and reports on effect sizes as well as key themes, examining the effectiveness of school-based interventions in reducing disproportionality in discipline practices based on student gender, race, ability status, and socioeconomic status. To expand on this work by Cruz and colleagues (2021) and adopt a more nuanced approach toward understanding the role of student *race* in discipline, a systematic review of the literature on interventions that address racism in disciplinary practices was conducted. This review included quantitative and qualitative studies to gain the most comprehensive understanding of interventions to address this issue. Furthermore, this review focused on the disparity based on *racial* identity rather than examining multiple intersecting identities as done by Cruz and colleagues (2021). In the data extraction, it was identified whether the intervention was culturally relevant, as this is crucial in reducing the race discipline gap (Edwards, 2021; Espelage et al., 2023; Gregory et al., 2021; Skiba et al., 2022).

With these questions and considerations in mind, the research questions guiding this systematic review are as follows:

1. What interventions exist to address racism in disciplinary practices in K-12 schools, and to what extent have those interventions been evaluated?
2. What types of interventions reduced racial disparities in discipline, and what research designs have been used to test the efficacy of those interventions?

This research is a critical step for summarizing the evidence for best practices for reducing racial disproportionality in discipline in K-12 schools and identifying the gaps that may need to be addressed to provide a comprehensive effort to reduce these disparities.

## Method

### Search Strategy

The protocol used in this study is informed by the Preferred Reporting Items for Systematic Reviews and Meta-Analysis (PRISMA) guidelines to search research databases, screen published studies, apply inclusion and exclusion criteria, and select relevant literature for review (see Appendix 1). The comprehensive electronic search of publications used the following databases: PsycINFO, ERIC, PubMed, Sociological Abstracts, and Scopus. The search was restricted to English-only studies and collected all database results published in the past 25 years (January 2000 to January 2025). The search terms addressed the main concepts of the search strategy: schools AND discipline AND intervention AND racism (see Appendix 2 for full search terms and syntax). Given the historical challenges with explicitly naming racism in US educational research, the search strategy for this review was purposefully designed to identify interventions that addressed racism in discipline disparities, even when the term “racism” was not used directly. As detailed in Appendix 2, a broad range of keywords, including related terms and concepts, was employed to capture studies that might use coded, indirect, or alternative language in place of explicitly naming racism. This approach ensured that interventions aimed at addressing racial disparities in discipline were included, regardless of whether the authors overtly named racism in their descriptions. After the full-text review, reference lists of included studies were assessed to identify any additional studies for inclusion, a process known as snowball sampling (Hiebl, 2023).

### Inclusion Criteria

To be included in this review, studies were required to be empirical and to evaluate interventions designed to address racism, racial disproportionality, or race-based disparities in exclusionary school discipline practices in K-12 schools. In these studies, the independent variable was defined as the implementation of an intervention, such as culturally relevant professional development, equity-focused policy changes, restorative practices, or bias reduction training targeted at reducing racial disparities or addressing racism within disciplinary processes. Thus, all included studies had a focus on race, racism, or racial disproportionality within their intervention goals. Both quantitative and qualitative studies were included, regardless of whether they were descriptive (e.g., describing an intervention) or experimental (e.g., implementing and reporting on the efficacy of an intervention). Furthermore, articles were required to be peer-reviewed, conducted in the USA, and published in English.

Articles were excluded if they (1) did not report on an intervention to address race, racism, or racial disproportionality; (2) did not report on disciplinary outcomes as the dependent variable; (3) did not report on an intervention (e.g., program, policy) concerning racism and discipline; (4) were not conducted in a K-12 school; (5) were conducted outside the USA; (6) were not published in English; (7) were not a peer-reviewed journal article; or (8) were a dissertation, review, editorial, opinion piece, or commentary.

### Study Selection

Covidence (2024), an online collaboration platform designed to streamline the process of systematic and other literature reviews, was utilized to organize the screening and selection of articles. The coding team consisted of three researchers: two senior scholars with expertise in violence prevention and systematic review methodology (Coders A and B), and one master’s-level researcher (Coder C) who was trained by the senior team. Coders A and B worked with a librarian to develop search terms and perform database searches. Coder C became involved in the screening phase and received structured training from the senior coders. All titles and abstracts were independently and blindly screened by two coders to assess their eligibility. Any discrepancies were discussed and resolved during team meetings through consensus. The same team members carried out a full-text screening of relevant articles to decide on their inclusion in the final sample. Following the full-text review, the team established standardized criteria and procedures for extracting information from the selected studies. Two team members conducted the initial data extraction, which was then reviewed for quality and accuracy by a third member. At each stage, the research team met to resolve any disagreements, ensuring all inclusion and exclusion decisions were made unanimously.

### Data Extraction

A data extraction procedure was developed for information from the articles that passed full-text review and met the inclusion criteria. The data extracted from the articles depended on the study design: descriptive (e.g., observational, case study) or experimental (e.g., quasi-experimental, randomized control trial). The following data were extracted from all studies: author, publication year, school level (elementary, middle, or high school), and geographical location (state or region represented). The research team also extracted details of the study design, including whether the study was quantitative (randomized control trial, observational research design, or quasi-experimental design) or qualitative (case study, ethnography, action research, or critical race theory). Furthermore, the unit of analysis (school, district, classroom, individual, state, or nation), the racial demographics of the students (e.g., majority students of color), and the audience (educators, school leaders (e.g., administrators, principals), school community, or students) were also extracted. For the purpose of this review, the term “educators” was used broadly to encompass all school personnel responsible for student instruction, supervision, or discipline within the K-12 setting. When possible, roles were differentiated in the data extraction process by identifying classroom teachers, aides, paraprofessionals, and mental health or behavioral staff (such as school counselors, psychologists, or social workers).

For the experimental studies, the sample size, percent female, and whether the intervention reduced racial disparities (yes or no) were extracted. To be coded “yes,” an intervention must report a comparison group. A disparity presupposes that one group is treated differently from another group, in this case, concerning school discipline practices (Okonofua & Eberhardt, 2015). Therefore, researchers who examined an intervention for one group of students, such as in the study by Gibson and colleagues (2019), were not considered successful in reducing racial disparities in discipline. Furthermore, studies were coded “yes” only when a reduction in disproportionality, defined as the gap in disciplinary outcomes between racial groups, was demonstrated, not merely a reduction in the risk index for a single group. For example, if an intervention reduced suspension rates for Black students but did not narrow the difference compared to White students, it was not coded as reducing disparities. This approach ensures that our review consistently identifies interventions that address comparative inequities in school discipline.

Drawing from the work by Skiba and colleagues (2022) outlining the widely adopted practices for school discipline, interventions were described using the following categories: Positive Behavioral Interventions and Supports (PBIS), Restorative Practices (RP), Social-Emotional Learning (SEL), Policy, Professional Development (PD), Learning Lab, and Other. The categories were developed after the initial search and screening process, but before data extraction. These categories were informed by Skiba and colleagues (2022) and were selected to guide the classification of interventions throughout the review. While the search and screening process was inclusive of all interventions addressing race disparities in school discipline, the predetermined categories provided a structured lens for data extraction and synthesis. In addition, a brief description of the intervention (see Tables 1 and 2) was provided. To operationalize the outcome variable (the discipline measure), the variable that the authors reported on (e.g., office discipline referral, expulsion) was extracted.

**Table 1:** Study characteristics of descriptive interventions (*n* = 21)

Author (year)	School level and location	Study design	Unit of analysis	Racial demographics	Intervention description	Outcome variable	Culturally relevant	Results
Amiot et al. (2020)	Middle school in UT	QUAL: CRT	School-wide professional development for educators	Majority students of color	CRT-informed intervention to challenge deficit framing toward students of color and reorient disciplinary actions	Equity audits; school data on discipline infractions and suspensions	Yes	Educators interrogate their racial biases in school discipline
Anyon et al. (2016)	Elementary, middle, and high school in CO	QUANT: ORD	District-wide RP for educators	Majority students of color	Voluntary staff trainings and recommendations to offer RP to students in conjunction with, or in place of, suspensions	ODRs; suspensions	No	Students who participated in RP in semester 1 were less likely to receive ODRs and suspensions in semester 2. The suspension gap between Black and White students persisted
Bal et al. (2014)	Elementary school in WI	QUAL: ethnographic case study	School-wide Learning Lab for the school community	Majority White school community	LL focused on providing opportunities for the school community to examine and address disproportionality in student outcomes	School culture	Yes	Implementation barriers include building inclusivity within a bureaucratic institution and shifting from a deficit to expansive discourse
Bal et al. (2019)	Middle school in WI	QUAL: case study	School-wide Learning Lab for the school community	Majority White and Black students	LL to design and address racial disproportionality in discipline through a culturally responsive school discipline system	Cultural responsiveness of school discipline	Yes	The process of creating LL was examined through needs, interests, and resources within the school community
Cornell et al. (2018)	Elementary, middle, and high school in VA	QUANT: ORD	State-wide policy for school leaders	Majority White students	Threat assessment teams	Suspensions; expulsions; school transfer; law enforcement action	No	No disparities among Black, Hispanic, and White students in OSS, school transfers, or legal actions
Curran (2016)	Elementary, middle, and high school in the USA	QUANT: ORD	National policy for school leaders	Majority White students	Zero-tolerance laws mandating expulsion across all 50 states	Exclusionary discipline; racial discipline gaps; student behavior	No	Laws are predictive of a 0.5-percentage-point increase in district suspension rates, more so for Black students than White students, contributing to the suspension gap
Fallo and Larwin (2024)	Elementary, middle, and high school in CA	QUANT: ORD	District-wide RP for school leaders	Racially diverse students	RP included affective statements, responsive circles, and restorative meetings	Suspensions; expulsions	No	Overall decline in expulsions and a decrease in the discipline gap between Caucasian and Black students. No change in suspensions
Green et al. (2024)	Elementary, middle, and high school in the USA	QUANT: ORD	State-wide policy for school leaders	Racially diverse students	Non-suspension and non-expulsion policies restrict the use of suspension and expulsion for certain student groups for particular behaviors	Suspensions; expulsions	No	In schools where the policy was implemented, Black students experienced lower rates of OSS but higher rates of expulsion
Gregory et al. (2024)	Elementary school; location NR	QUAL: case study	School-wide RP for educators	Racially diverse students	RP implemented using a tiered training approach, including leadership-focused trainings, staff-wide equity activities, and individualized intensive training supports	School culture	Yes	Equity-centered values of school leaders and capacity and support for RP allowed for harm reduction, nurturing relationships, and equitable learning environments
Hashim et al. (2018)	Elementary, middle, and high school in CA	QUANT: ORD	District-wide policy for school leaders	Racially diverse students	Suspension bans for willful defiance and programs to train educators in restorative justice to address disproportionate suspension rates	Suspensions	No	Overall decline in suspension rates; suspension gaps between Black and non-Black students persisted
Heidelburg et al. (2022)	Elementary and middle school on the US East Coast	QUANT: ORD	District-wide PBIS for school leaders	Majority Black students	Analysis of SWPBIS implementation and ODRs for Black students	ODRs	No	No linear relationship was found between SWPIBS implementation and reduction of ODRs among Black students
Kim et al. (2024)	Elementary, middle, and high school in CA, CO, and NY	Mixed methods	School-wide RP for educators	Majority students of color	RP is used by school leaders and educators to replace exclusionary discipline and integrate RP in school policy/code of conduct	Suspensions	No	RP led to lower suspensions for students overall
Ko et al. (2022)	High school in WI	QUAL: case study	School-wide Learning Lab for school community	Racially diverse students	Cultural–historical activity theory-based intervention where school community members analyze systemic challenges in the community and develop context-specific solutions	Types of transformative agency utilized by participants	Yes	Participants developed a culturally responsive behavioral support system to address racial disproportionality in discipline outcomes
Mansfield et al. (2018)	High school in VA	QUANT: ORD	School-wide RP for school leaders	Racially diverse students	*SaferSanerSchools* is a comprehensive, multitiered, preventative, and responsive RP	Suspensions	No	Reduction in the race discipline gap with regard to suspensions
Mawene et al. (2024)	High school in WI	QUAL: CRT	School-wide Learning Lab for the school community	Majority White students	*ThirdSpace* is an Indigenous LL to develop localized and community-driven solutions to racial disparities	Cultural responsiveness of school discipline	Yes	Members questioned the race-neutral approach to interpreting data, explored dystopian scenarios, revitalized American Indian ways of knowing, and developed a vision for a new educational system
Stephens (2021)	Elementary school location NR	QUAL: action research	Classroom-wide SEL for the school community	Black and Hispanic students	Three-phase student-led effort on the impact of a restorative justice–inspired responsive SEL program	Student response to SEL program; OSS	Yes	Violent classroom disputes and OSS decreased
Tobin and Vincent (2011)	Elementary, middle, and high school in CO, IL, MD, and MI	QUANT: ORD	School-wide PBIS analysis for educators	Majority White students	Analysis of disproportionate exclusion of Black students using educators’ self-reported implementation of SWPBIS and RRI	Relative Rate Index (RRI)	No	SWPBIS strategies were associated with reductions in disproportionate exclusionary discipline
Vincent and Tobin (2011)	Elementary, middle, and high school in CO, IL, MD, MI, OH, OR, & SC	QUANT: ORD	School-wide PBIS analysis for the school community	Majority students of color	Analysis of SWPBIS implementation, student ethnicity, and ODR	OSS; Effective Behavior Support (EBS)	No	SWPBIS was associated with decreased exclusions in elementary and high schools. Black students remained overrepresented in exclusions
Vincent et al. (2011)	Elementary school in IL, OR, CO, IA	QUANT: ORD	School-wide PBIS analysis for the school community	Majority students of color	Analysis identifying patterns of ODR with SWPBIS	ODR	No	SWPBIS schools had a smaller race discipline gap compared with non-SWPBIS schools
Wang (2022)	High school in CA	QUANT: ORD	District-wide policy for school leaders	Racially diverse students and educators	Willful Defiance Suspension Ban (WDB)	OSS	No	OSS decreased for willful defiance, but not for other infractions; OSS increased among Black students
Zakszeski et al. (2021)	Elementary, middle, and high school in the Mid-Atlantic U.S.	QUANT: ORD	District-level SWPBIS analysis for the school community	Majority students of color	Analysis of SWPBIS by student race and ODR	ODRs; risk indices; risk ratios; risk differences	No	ODRs remained overrepresented among Black and Hispanic students

*CRT* critical race theory, *PBIS* positive behavioral intervention and supports, *RP* restorative practices, *SEL* social–emotional learning, *OSS* out-of-school suspensions, *ISS* in-school suspensions, *ORD* observational research design

**Table 2:** Study characteristics of experimental interventions (*n* = 27)

Author (year)	School level and location	Unit of analysis	Racial demographics	Intervention description	Outcome variable	Reduced race disparity	Culturally relevant	Percent female	Sample size	Results
***Quasi-experimental designs (n = 14)***										
Anderson and McKenzie (2023)	Elementary school in AR	District-wide policy for school leaders	Majority White students	Policy enforcing limits on exclusionary discipline	Infraction types and frequency	No	No	NR	NR	The policy reduced the risk of suspension or expulsion for all students. Black students had the least reduction in risk compared to other racial groups
Ash et al. (2023)	Elementary, middle, and high school in the USA	Individual-level SEL for educators	Majority White educators	Online survey to assess mindfulness practices and the relationship to anti-Black bias and disciplinary decisions among educators	Adapted version of the Disciplinary Practices Survey	No	No	61.5%	179	Educator self-reported mindfulness moderated responses to a disciplinary action as a function of student-identified race
Cook et al. (2018)	Elementary school in the western U.S.	School-wide PBIS for educators	Majority students of color	*Greet Stop Prompt* (GSP) aims to mitigate exclusionary discipline through classroom management, self-regulation, and empathetic responses to student behavior	Relative risk ratios	Yes	No	76%	40	GSP led to reductions in risk ratios. The likelihood of Black students receiving an ODR was cut by two-thirds
Davison et al. (2022)	Elementary, middle, and high school in the western U.S.	District-wide restorative practice for school leaders	Majority White students	Difference-in-difference estimates of the effects of RP on student discipline based on race	Suspensions	No	No	49.7%	7282	Students in RP schools experienced a decline in suspension rates. However, disciplinary outcomes for Black students were largely unchanged; racial disproportionality widened
Gibson et al. (2019)	Middle school in TN	School-wide SEL for students	Black boys	Culturally responsive group intervention to improve behavior and increase social and emotional skills for Black boys	ODRs	No	Yes	0%	8	73.68% decrease in ODRs among the group of Black boys
Gion et al. (2020)	Elementary and middle school in the Pacific Northwest U.S.	Classroom-wide behavioral intervention for educators	Racially diverse students	Classroom-based behavior systems intervention to increase cultural responsiveness and address racial disparities in teacher behavior	Praise and reprimands	Yes	Yes	75%	4	Increase in praise for all groups and decrease in reprimands, more so for Black students than other racial groups
Knochel et al. (2022)	Elementary school in the Southeast U.S.	Classroom-wide professional development for educators	Majority students of color	Behavior-specific praise (BSP) with self-monitoring and written performance feedback	BSP and reprimands	No	No	100%	4	Overall reduction in reprimands; disparities in BSP across student racial groups persisted
Lee et al. (2021)	Elementary and middle school in GA	School-wide PBIS for school leaders	Majority students of color	A multitiered system of support for school-wide expectations and student behavior	ISS; OSS; expulsions; school transfers; law enforcement action	Yes	No	51.7%	1403	Reduction in exclusionary discipline, especially for Black students, apart from arrests
Lo and Cartledge (2006)	Elementary school in the Midwest U.S.	Individual-level behavioral intervention for students	Black students and White educators	Behavioral intervention plans (BIP) include skill training, differential reinforcement, and self-monitoring of behavior for Black boys	Off-task behavior	No	No	0%	4	BIP reduced off-task behavior to a level comparable to their peers
McIntosh et al. (2021b)	Elementary, middle, and high school in the Southeast U.S.	School-wide professional development for school leaders	Majority students of color	4 days of PD for school leaders focused on PBIS to improve equity in school discipline	Exclusionary discipline	No	Yes	NR	NR	Improvements in school exclusionary discipline when compared to comparable non-participating schools
Okonofua et al. (2022)	Middle school in the Southeast U.S.	Classroom-wide SEL for educators	Majority students of color and White educators	“Empathic mindset” intervention to refocus selected teacher perspectives to understand student behavior	ODRs	Yes	No	79%	66	Reduced suspension rates for Black and Hispanic students compared to their peers
Pimentel-Mannan et al. (2024)	Middle school in the Pacific Northwest U.S.	School-wide professional development for educators	Racially diverse students	*Inclusive Skill-Building Learning Approach* is a restorative and instructional alternative to exclusionary discipline practices	ODRs; ISS; OSS	No	No	NR	1084	Reduction in overall discipline measures and risk indices for ODRs for students of color; disparities persisted
Rila et al. (2022)	High school in the Midwest U.S.	School-wide behavioral intervention for educators	Majority White students	Visual Performance Feedback (VPF) for teachers’ equitable delivery of BSP and student reprimands	BSP; reprimands; ODRs	No	No	53%	99	VPF decreased the frequency of reprimands and increased BSP for participating teachers
Scott and Collins (2024)	High school in the Midwest U.S.	Classroom-wide SEL for students	Racially diverse students	*Fix Your Crown, Queen* is a culturally enriched SEL curriculum for Black girls	ODRs	No	Yes	100%	5	Three out of five participants had a decrease in ODRs
***Randomized controlled trials (n = 13)***										
Austin et al. (2024)	Elementary school in the Southeast U.S.	School-wide PBIS for educators	Racially diverse students	A series of equity-centered professional development sessions on PBIS strategies	ODRs	Yes	Yes	88%	441	Reduction in racially disproportionate ODRs for Black students at treatment schools
Borman et al. (2022)	Middle school in WI	District-wide SEL for students	Majority White students	A self-affirmation exercise asking students to identify values and why they are important to them	Suspensions	Yes	No	50%	2149	Reduction in the Black–White suspension gap by 67%
Bradshaw et al. (2018)	Elementary and middle school in MD	School-wide professional development for educators	Majority students of color	A coaching approach that is utilized as an element of the *Double Check* cultural responsivity and student engagement model	ODRs; relative risk ratios	No	Yes	85.4%	158	Teachers who received the coaching decreased the use of ODRs for Black students
Debnam et al. (2024)	Middle school in MD	School-wide professional development for educators	Majority students of color	*Double Check* is a model to improve cultural responsiveness and engagement	OSS	No	Yes	76.7%	352	Significant improvement in teacher self-efficacy to use culturally responsive practices; no significant changes in overall or disproportionate suspension rates
Goyer et al. (2019)	Middle school in the Northeast and Western U.S.	School-wide SEL for students	Majority students of color	Social-belonging, values-affirmation, and growth-mindset intervention intended to promote identity safety for negatively stereotyped boys	Discipline citations	Yes	No	49.6%	669	Reduced citations of negatively stereotyped boys; closed the disparity between Black and White boys over 7 years
Gregory et al. (2014)	Middle and high school in the Southeast U.S.	Classroom-wide professional development for educators	Majority students of color	*My Teaching Partner-Secondary* (MTP-S) provides teachers with ongoing personalized coaching and feedback to improve teacher–student interactions	ODRs	Yes	No	52%	82	Black students had a similarly low probability of receiving ODRs compared with students of other racial groups
Gregory et al. (2016)	Middle and high school in VA	Classroom-wide professional development for educators	Majority Black students	*My Teaching Partner-Secondary* (MTP-S) provides teachers with ongoing personalized coaching and feedback to improve teacher-student interactions	ODRs	Yes	No	65%	86	Reduced the racial discipline gap in ODRs
Gregory et al. (2019)	Middle and high school in the Southeast U.S.	Classroom-wide professional development for educators	Majority Black students	*My Teaching Partner-Secondary* (MTP-S) provides teachers with ongoing personalized coaching and feedback to improve teacher–student interactions	ODRs	Yes	No	NR	58	Reduced the racial gap in ODRs; sustained 1 year post-intervention
Huang et al. (2023)	Elementary, middle, and high school in the Northeast U.S.	District-wide restorative practice for educators	Majority Black students	*Whole School Restorative Practices Project* is a restorative practice model that centers racial equity in SEL	OSS	No	No	50%	5878	No suspension differences found among students in the intervention and control schools; reductions in OSS for students with previous suspensions
McIntosh et al. (2020)	Elementary, middle, and high school in the Pacific Northwest U.S.	School-wide discipline information system for school leaders	Black and White students	Disciplinary equity reports	District discipline data	No	No	NR	NR	No meaningful change in disciplinary equity
McIntosh et al. (2021a)	Elementary school in the Southeast U.S.	School-wide PBIS for the school community	Black students	Multicomponent, equity-focused SWBPIS	ODRs	Yes	Yes	NR	9600	Decrease in racial disparities in discipline and ODRs
Okonofua et al. (2020)	Elementary, middle, and high school in the Southern U.S.	Individual-level psychological intervention for educators	Majority White educators	In Bias Consequence Alleviation (BCA), educators report how they would react in hypothetical situations based on perceived student racial identity	Consequences of educator self-reported bias and perceptions of student behavior	Yes	No	88.6%	246	BCA reduced racial inequality in teachers’ hypothetical discipline of Black and White students
Vincent et al. (2023)	High school in the Pacific Northwest	School-wide professional development for school leaders	Majority White students	Professional development focused on integrating RP into multi-tiered systems of support	Student perceptions of discipline	No	No	NR	16	Students in intervention schools had improved perceptions of disciplinary consistency and equity compared to students in control schools (not statistically significant)

*NR* not reported, *PBIS* positive behavioral intervention and supports, *RP* restorative practices, *SEL* social–emotional learning, *OSS* out-of-school suspensions, *ISS* in-school suspensions

Finally, the research team indicated whether each intervention was culturally relevant. To determine this, the team systematically reviewed each article for explicit descriptions as well as substantive indicators of culturally relevant, asset-based, or culturally affirming frameworks, such as culturally relevant, responsive, or sustaining pedagogies, beyond simply acknowledging racial disparities. This included identifying whether interventions incorporated practices aligned with culturally relevant pedagogy (e.g., integration of students’ cultural backgrounds, community and family engagement, promotion of cultural competence among educators, or reference to foundational theorists such as Ladson-Billings), even when specific terminology such as “culturally relevant” or “culturally responsive” was not used. Interventions were coded as “yes” if cultural relevance was described as a primary aspect of the intervention, either through explicit language (e.g., equity focused, culturally relevant, culturally responsive, or race conscious) or through detailed intervention components and theoretical underpinnings demonstrating these principles. Authors who discussed cultural relevance only as a secondary goal, afterthought, implication, or future direction were coded as “no.” This comprehensive approach was intended to capture interventions grounded in culturally relevant principles, even if not labeled explicitly as such by study authors.

These data extraction procedures were purposely designed to address our research questions regarding both the existence and evaluation of interventions targeting racism in school disciplinary practices. By extracting information on intervention types, implementation context, participant demographics, research design, and measured outcomes (including reductions in racial disparities), our process enabled a comprehensive synthesis of not only what interventions have been implemented, but also the extent and rigor with which they have been empirically evaluated in K-12 settings. Additionally, coding for cultural relevance and intervention efficacy allowed us to analyze which approaches were most effective in reducing disparities, directly informing our inquiry into intervention types and their impact across varied research designs.

## Results

**Fig. 1:**
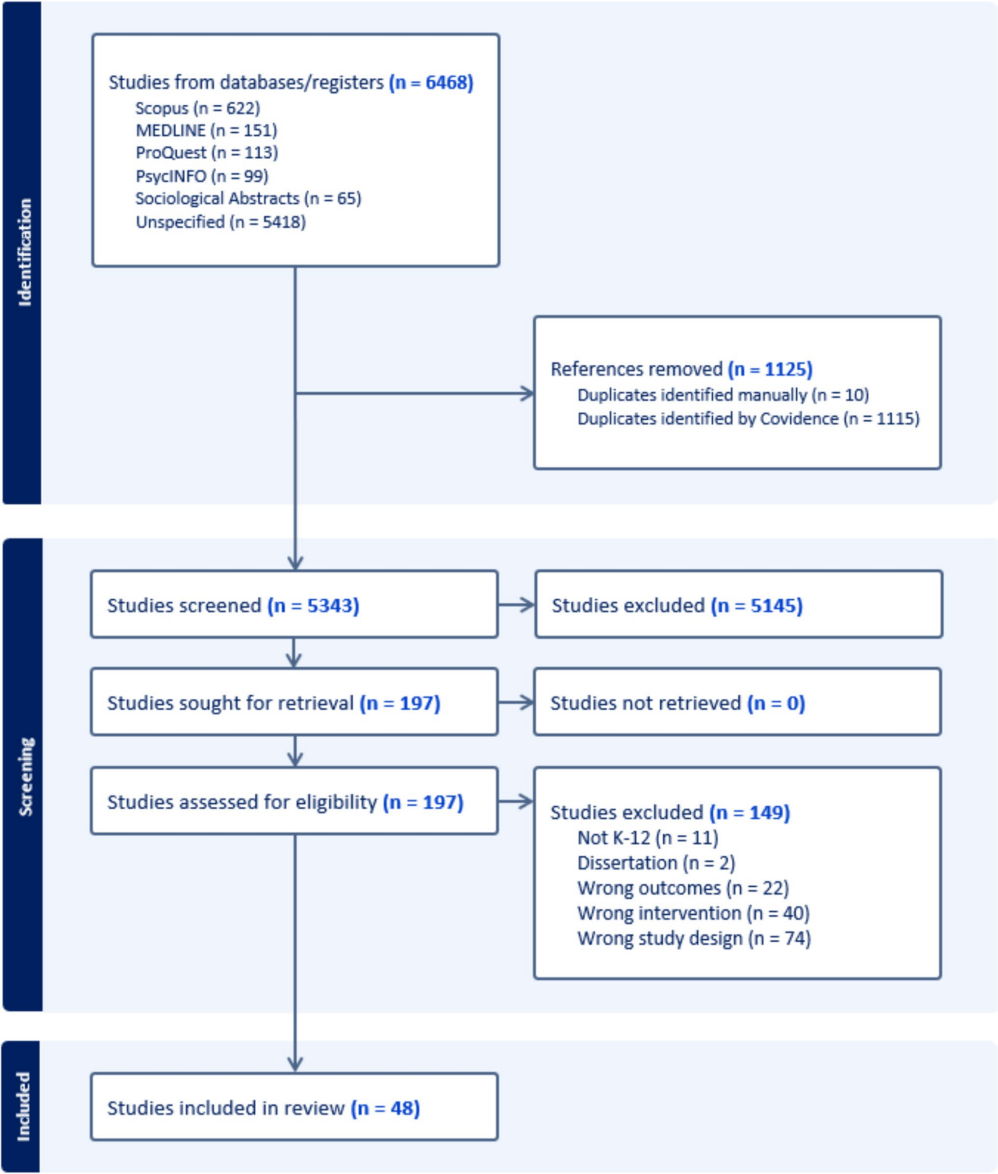
PRISMA flow chart for systematic review process

Figure 1 depicts the systematic review process. The initial database search identified 6468 articles, of which 1125 were identified as duplicate records and removed. Next, 5343 records were examined during the initial title/abstract screening, of which 5145 were excluded due to not meeting the identified inclusion/exclusion criteria. A total of 197 records were examined during full-text review. Of these records, 149 were excluded due to not meeting the established inclusion/exclusion criteria. In total, the final sample consisted of 48 studies that were included in the review. Below, these 48 studies are reported on and discussed based on whether they were descriptive or experimental research designs. Furthermore, a subsample of experimental interventions that reduced racial disparities is critically analyzed. Studies were coded as “reducing disparities” if they demonstrated a narrowing of racial gaps in exclusionary discipline outcomes between groups, even if disparities were not fully eliminated. The goal of this coding was to identify interventions associated with a measurable reduction in race-based disciplinary disproportionality, not the total eradication of disparities.

### Descriptive Studies

Twenty-one articles reported an intervention description, as detailed in Table 1. Among these descriptive studies, one of the most frequent interventions reported was Positive Behavioral Interventions and Supports (PBIS; *n* = 5). Of the five studies on PBIS, the intended audience varied from the school community (*n* = 3), educators (*n* = 1), and school leaders (*n* = 1). For example, Zakszeski and colleagues (2021) examined ODR trajectories using secondary district-level data from 27 schools that had implemented school-wide PBIS over 3 years. They found that ODRs among Black and Hispanic students remained overrepresented across the years of PBIS implementation (Zakszeski et al., 2021). Another intervention described was policies (*n* = 5), all of which were intended for school leaders. The studies on Learning Lab (*n* = 4) and social-emotional learning (*n* = 1) described the audience as the whole school community. Articles on restorative practices (*n* = 5) described the intended audience as educators (*n* = 3) and school leaders (*n* = 2), while the article on staff professional development (*n* = 1) described educators as the audience.

All the included descriptive quantitative studies use observational research designs (*n* = 13). While these studies provide valuable information about interventions and their potential to help address this problem, observational research designs do not yield causal results, thus precluding conclusions about program effectiveness in reducing disparities (Campbell & Stanley, 2011). The seven qualitative studies included an ethnographic case study (*n* = 1), case study (*n* = 3), critical race theory (*n* = 2), and action research (*n* = 1) approach. While these qualitative studies are limited in generalizability due to the in-depth analysis of one specific school (or grade, as in the article by Stephens, 2021), the findings may be insightful or applicable in developing and implementing equity-focused interventions in other contexts. For example, in the ethnographic case study by Bal and colleagues (2014), they discuss the process of engaging local stakeholders in implementing *Learning Lab*, a school-wide equity-focused adaptation of PBIS, in an elementary school in Wisconsin to address racial disparities in student outcomes, including discipline. They provide critical considerations for the formation of equity-focused interventions that provide systemic transformation, including “how families’ cultural practices, histories, and goals are included; and ultimately how such a knotworking builds the institutional capacity for sustained coalitions among schools, families, community-based organizations, and local educational agencies” (p. 337). Thus, the value of these qualitative studies is not to be understated.

Interestingly, the researchers in all seven qualitative studies described the interventions as culturally relevant in their respective studies. The researchers described none of the 13 quantitative (observational) studies as culturally relevant, but they did examine racial disparities. Most interventions among these descriptive studies took place across all three school levels (elementary, middle, and high; *n* = 10), with others focusing on the elementary (*n* = 4), elementary and middle (*n* = 1), middle (*n* = 2), and high (*n* = 4) school levels. The units of analysis included school-wide (*n* = 11), district-wide (*n* = 6), classroom-wide (*n* = 1), state-wide (*n* = 2), and nationwide (*n* = 1). In addition, the studies represented a wide range of geographic locations in the USA where these interventions take place (see Table 1).

### Experimental Studies

Twenty-seven studies reported an experimental methodology to evaluate the intervention (see Table 2). The most commonly reported interventions among these experimental studies were focused on staff professional development (*n* = 9). Among the nine studies on staff professional development, the intended audience included educators (*n* = 7) and school leaders (*n* = 1). Knochel and colleagues (2022), for example, examined the use of behavior-specific praise with self-monitoring and performance feedback among four female educators in an elementary school in the Southeast U.S. Using a multiple baseline design, they found that disparities in educators’ use of behavior-specific praise persisted across student racial groups with an overall reduction in reprimands (Knochel et al., 2022). These findings may be attributed to the lack of focus on addressing implicit bias and racial inequities as part of the intervention.

Another type of intervention described was social-emotional learning (SEL; *n* = 6). The intended audience of the six articles on SEL included students (*n* = 4) and educators (*n* = 2). Authors of articles on PBIS (*n* = 4) described the audience as educators (*n* = 2), school leaders (*n* = 1), and the broader school community (*n* = 1). The audience for articles on RP (*n* = 2) included educators (*n* = 1) and school leaders (*n* = 1), and the audience for the article on a policy was school leaders (*n* = 1). Articles with interventions that did not fit into one of these categories were described as other (*n* = 5), and these interventions were for educators (*n* = 3), students (*n* = 1), and school leaders (*n* = 1).

Most interventions among experimental studies occurred at the elementary school level (*n* = 6), followed by those at all three grade levels (elementary, middle, high; *n* = 5). Other school levels in these experimental studies include interventions at elementary and middle (*n* = 3), middle (*n* = 4), middle and high (*n* = 3), and high (*n* = 1) school levels. The units of analysis included district-wide (*n* = 4), school-wide (*n* = 9), classroom-wide (*n* = 6), and individual-level (*n* = 3).

The researchers in all 27 studies used quantitative methods; 14 articles used quasi-experimental designs (QED), and 13 used randomized control trials (RCTs). Of the 14 QEDs, one article had an intervention that both reduced racial disparities and was culturally relevant (Gion et al., 2020); two articles had interventions that did not mitigate racial disparities but were culturally relevant (Gibson et al., 2019; McIntosh et al., 2021b); four articles had interventions that reduce racial disparities but were not culturally relevant (Cook et al., 2018; Lee et al., 2021; Okonofua et al., 2022; Scott & Collins, 2024); and seven articles had interventions that neither reduced racial disparities nor were culturally relevant (Anderson & McKenzie, 2023; Ash et al., 2023; Davison et al., 2022; Knochel et al., 2022; Lo & Cartledge, 2006; Pimentel-Mannan et al., 2024; Rila et al., 2022).

Of the 13 RCTs, two articles had an intervention that both reduced racial disparities and was culturally relevant (Austin et al., 2024; McIntosh et al., 2021a); two articles had an intervention that did not mitigate racial disparities but was culturally relevant (Bradshaw et al., 2018; Debnam et al., 2024); six articles had an intervention that reduced racial disparities but was not culturally relevant (Borman et al., 2022; Goyer et al., 2019; Gregory et al., 2014, 2016, 2019; Okonofua et al., 2020); and three articles had an intervention that neither reduced racial disparities nor was culturally relevant (Huang et al., 2023; McIntosh et al., 2020; Vincent et al., 2023). In addition, the studies represented a wide range of geographic locations in the USA where these interventions take place (see Table 2).

### Reduction in Racial Disproportionality

Twelve of the 27 experimental studies report results of an intervention that reduced racial disparities in school discipline. Researchers suggest that interventions tailored to those enacting the discipline practices will most likely reduce disparities as opposed to interventions for students (Skiba et al., 2022; Tobin & Vincent, 2011). Therefore, the following sections are focused on a subsample of interventions designed for educators (*n* = 8) and school leaders (*n* = 1).

Of the nine articles with interventions for educators and school leaders, there were five randomized control trials (RCTs) and four quasi-experimental designs (QEDs). For example, Okonofua and colleagues (2020) used an RCT to evaluate an individual-level psychological intervention for educators designed to alleviate racial bias in school discipline practices. While the authors report that the intervention reduced racial inequality in teachers’ discipline of Black and White students, the intervention involved a host of hypothetical scenarios presented to participants on a computer screen. While the preliminary evidence is promising, researchers may consider replicating this study in a real-world classroom setting to increase confidence in the findings. Furthermore, the majority of the participants in this study were white educators, limiting the generalizability of these findings. However, data suggest that 80% of public school teachers identify as white (U.S. Department of Education, 2022).

These intervention types included professional development (*n* = 3), PBIS (*n* = 3), SEL (*n* = 1), and other (psychological and behavioral interventions; *n* = 2). The three articles on professional development for educators all tested the same intervention, *My Teaching Partner*, in different middle and high school samples using RCTs (Gregory et al., 2014, 2016, 2019). In all three studies, Gregory and colleagues (2014, 2016, 2019) found that the intervention reduced the race discipline gap in ODRs for Black and White students. While these findings are promising, a few areas for future research have been identified.

First, the sample sizes for these studies are small (*N* = 58 to 86), limiting the confidence in the results. Future researchers may consider replicating this program evaluation with a larger sample of educators. Second, these studies were conducted in middle and high schools in the Southeast U.S., limiting the generalizability of the findings. Researchers suggest the race discipline gap is ubiquitous across all school levels and geographic regions. Thus, future researchers may consider replicating this study in other contexts. Third, while race disparities exist in all school discipline practices, including suspensions, expulsions, arrests, and school transfers, the outcome for all three studies is limited to only ODRs. While ODRs are informative for documenting student behavioral issues, future researchers may consider examining the effect of *My Teaching Partner* on other discipline outcomes. Finally, while researchers demonstrate that the race discipline gap affects Black and Hispanic students, these studies only examine the disparities between Black and White students. Future researchers may consider replicating this study with Hispanic students to test the effect of the intervention in addressing the disparity for these students.

### Culturally Relevant Interventions

Three articles detailed the results of a culturally relevant intervention that reduced race disparities in school discipline with an experimental methodology. Gion and colleagues (2020) utilized a concurrent multiple-baseline, single-case design across four elementary and middle school teachers to evaluate a classroom-based behavior systems intervention to increase cultural responsiveness and address racism in teacher behavior. The intervention is an adaptation of the evidence-based *Classroom Check-Up* model, which includes tools for educators to reflect on their implicit biases and coaching with visual performance feedback on the inequitable treatment of students. They found that implementing the intervention was associated with increased praise and decreased reprimands for all students, the most substantial being for Black students. Thus, Gion and colleagues (2020) concluded that changing teachers’ use of praise and reprimands may help to increase racial equity in schools. The scale of this intervention, however, is limited to the classroom. It does not consider the larger school context vital for systemic change and sustaining intervention effects beyond the classroom (Engeström & Sannino, 2011; Wiltsey Stirman et al., 2012).

McIntosh and colleagues (2021a) conducted a randomized control trial to see if multicomponent, equity-focused, school-wide PBIS would reduce the rate of ODRs between Black students and their peers in 13 elementary schools. They used a framework called *ReACT* (*Racial equity through Assessing data for vulnerable decision points, Culturally responsive behavior strategies, and Teaching about implicit bias and how to neutralize it*) to leverage the intervention. McIntosh and colleagues (2021a) found that intervention schools had significant decreases in rates of ODRs for Black students, while the control schools had minimal change. They conclude that equity-focused, multi-tiered systems of support implemented with fidelity can help reduce racial disparities in school discipline. This finding has limited generalizability because they examined ODR rates for Black students in a sample of eight elementary schools from one school district. Thus, these findings cannot be generalized to students of other marginalized racial backgrounds. Furthermore, additional research is needed to examine the effects of the *ReACT* framework on other longitudinal outcomes, such as academic achievement, given the adverse effects of discipline disparities on academic achievement (Morris & Perry, 2016; Noltemeyer et al., 2019).

Lastly, Austin and colleagues (2024) examined the effectiveness of an equity-centered positive behavioral interventions and supports professional development intervention using a randomized control trial. The authors also used the *ReACT* framework to assess their intervention. They found that the racial disproportionality of office discipline referrals between Black students and students of other racial groups decreased at the treatment schools. Austin and colleagues (2024) also found that the implementation and feasibility of the intervention were acceptable for the teachers in the study. Unfortunately, the study only took place within one school district, limiting the generalizability of their findings.

## Discussion

The present findings suggest that, despite the ubiquity of racial disparities in school discipline, there are limited rigorous evaluations of interventions designed to address racial disproportionality in school discipline. The present study builds on Cruz and colleagues’ (2021) review because it included 33 articles published after May 2019, the last year of their search (although there were nine overlapping articles). Moreover, the present review included articles that utilized qualitative research designs to ensure complete coverage of the interventions that have been published. In contrast, Cruz and colleagues (2021) restricted their search to only articles with quantitative research designs. Nevertheless, there were few studies with rigorous evaluation designs (i.e., quasi-experimental, randomized control trial) focused on reducing the race discipline gap, demonstrating the need for additional research to establish the generalizability of intervention effects and best practices for reducing discipline disparities. The limited evaluation research on racism and school discipline practices leaves school leaders with minimal guidance on what evidence-based best practices may help reduce the race discipline gap in their schools.

There are several recommendations from this review that may help provide some ideas for addressing the race discipline gap in practice. Culturally relevant professional development for educators and school leaders is likely more effective than programs (e.g., social-emotional learning) for students. Components of culturally relevant professional development for educators may include opportunities to challenge implicit biases, learn about the history of structural racism in education, and commit to increasing equitable discipline practices and classroom management strategies. Existing strategies in schools, such as Positive Behavioral Intervention and Supports (PBIS), Restorative Practices (RP), and Social-Emotional Learning (SEL), may help reduce race disparities in discipline when the principles are valued by the school community and implemented with an equity focus. For example, practitioners implementing equity-focused (transformative) SEL in schools provide opportunities for youth to use their voices to create positive change and developmentally appropriate activities to engage students and adults in examining how social issues, such as racism, impact society (Jagers et al., 2019).

It is important to acknowledge that many PBIS frameworks include elements of restorative practices and SEL across multiple tiers (Gregory et al., 2021). In this review, interventions were coded based on the primary focus as described by the study authors and the terminology used in the intervention’s documentation or evaluation. Where PBIS programs incorporated restorative or SEL components, those additional features were noted (see Tables 1 and 2), though the intervention was categorized according to the overarching framework named by the authors. This approach strives to maintain clarity and consistency while recognizing the complexity and integration of multi-tiered intervention systems. Future reviews may consider more nuanced coding strategies to reflect the multi-component and evolving nature of school-based interventions.

The findings of this study suggest that when traditional programs like PBIS, SEL, and RP are implemented with an intentional focus on race and cultural relevance, they may mitigate race-based disparities in school discipline. These findings are consistent with previous research that successful school interventions for reducing race disparities value diversity in student cultures rather than those designed to assimilate students into the majority culture. Interventions that are not culturally relevant are unlikely to mitigate race disparities in school discipline because they fail to honor cultural differences among youth, allowing disparities to persist (Anyon et al., 2016; Curran, 2016; Hashim et al., 2018; Knochel et al., 2022; Zakszeski et al., 2021) and sometimes even widen (Davison et al., 2022). Interventions addressing root causes of inequalities, such as structural and personal biases, may be most effective at mitigating the race discipline gap.

While addressing the root causes of racial discipline disparities, such as structural and interpersonal bias, is crucial for achieving equity, it is important to acknowledge the significant challenges involved in developing and implementing interventions that directly confront these issues. Interventions that seek to address structural racism and implicit bias often require fundamental shifts in school culture, policies, and individual mindsets, which can encounter resistance due to differing beliefs, institutional constraints, and limited resources. Furthermore, the feasibility and acceptability of these interventions may vary across school communities, with some educators or administrators potentially reluctant to engage in practices perceived as controversial or demanding substantial change. As such, even evidence-based interventions may face barriers to adoption, fidelity, and sustainability in real-world school settings. Future research and implementation efforts may consider these challenges and explore ways to support schools and educators in adopting and maintaining interventions that effectively address both interpersonal and structural contributors to discipline disparities.

The field has yet to make significant progress in replicating empirical research on interventions that reduce the race discipline gap. While researchers demonstrate that disproportionate discipline affects Hispanic students (Crosse et al., 2022), no randomized control trials in this review examined this disparity among Hispanic students. Furthermore, the three evaluations of the *My Teaching Partner-Secondary* (MTP-S) intervention by Gregory and colleagues (2014, 2016, 2019) had relatively small sample sizes of students (*N* = 58 to 82) and were specific to the middle and high school levels. In addition, these researchers evaluated the same intervention approach (MTP-S), but they replicated their findings across studies and settings. Future research is needed to address these gaps in the literature to develop a comprehensive, holistic evidence base for school-based interventions that reduce the race discipline gap.

### Limitations

This study has some limitations. First, only studies that were peer reviewed and published in English were included, excluding articles published in languages other than English, as well as gray literature or other work of less scientific rigor. School districts or other entities may complete internal evaluations of their efforts to mitigate the race discipline gap without disseminating findings. Because this review only included peer-reviewed literature, unpublished program evaluations were not included. This presents the risk of publication bias, as studies demonstrating significant or positive effects are more likely to be published in peer-reviewed journals, which may lead to an overestimation of intervention effectiveness and limit the generalizability of the present findings.

Second, although qualitative and quantitative research articles were included to gain the most data about race discipline gap intervention descriptions and evaluations, the inclusion criteria introduced heterogeneity into the rigor and, thus, the quality of the studies included in this review. As a result, the final sample included studies that could not address program effectiveness and may not be informative to guide school policy. Nevertheless, including all types of designs provided an opportunity to describe different approaches to reduce racism in school disciplinary actions.

Third, the data extraction for this study did not include whether training or coaching was delivered by individuals internal to the school (such as staff or district personnel) or by external trainers, consultants, or researchers. This distinction is particularly relevant for interventions seeking to foster culturally responsive practices, as the cultural familiarity and contextual knowledge of the trainer or implementer may influence both the acceptance and effectiveness of the intervention. Future researchers would benefit from systematically examining whether implementers are internal or external, the degree of their familiarity with the specific school community, and the potential implications for intervention fidelity, cultural relevance, and sustainability. Collecting and analyzing such data could inform best practices for implementing and scaling culturally relevant disciplinary interventions.

Fourth, a notable limitation of this review is the variability in how studies defined and reported the roles of “educators” participating in interventions. While efforts were made to distinguish among classroom teachers, aides, paraprofessionals, and mental health or behavioral staff, many studies used the term “educators” broadly or did not specify participant roles. As a result, there may be ambiguity in interpreting which school personnel were directly involved in or impacted by the interventions. Future researchers may consider specifying the roles of participating staff to better understand the differential effects of interventions across educator roles.

Finally, geographic locations of the interventions included in this review can be found in Tables 1 and 2; however, a systematic analysis of the impact of geographic context on the existence or effectiveness of racism interventions is beyond the scope of this study. Future researchers may further examine how geographic location may contribute to the development and implementation of interventions addressing the race discipline gap.

## Conclusion

Exclusionary school discipline practices can lead to a host of negative consequences for all students, especially for students of color, as they are disciplined at higher rates than their White peers for similar behavioral infractions. This review of the literature demonstrates that the field has made little progress in establishing an evidence base for interventions to reduce race disparities in school discipline since Cruz and colleagues published their best-evidence synthesis in 2021. Based on their findings, Cruz and colleagues (2021) concluded that efforts to mitigate the race discipline gap have lacked data on “the extent to which embedded structural and personal biases affect intervention effectiveness” (p. 417). The present findings support this conclusion despite the broader inclusion criteria and additional studies. Although additional culturally relevant interventions were identified, rigorous evaluation of them remains a significant limitation for informing school policy. Only three interventions were tested with an experimental design that reduced the race discipline gap. These interventions include professional development programs focused on building cultural competence and equity-mindedness among teachers and administrators, interventions providing ongoing coaching and feedback to address implicit bias, and data-monitored systems that encourage reflective practice and accountability in disciplinary decisions. Given these considerations, there is a need for interventions that challenge the biases of those with the authority to enact discipline measures. Interventions designed for students may not be most effective at reducing disparities, given that student behavior is not the root cause of racial disparities. Future researchers may consider tailoring interventions to school leaders and educators whose actions ultimately perpetuate or mitigate disparities.

## Appendix 1

**Table:** PRISMA checklist

Section and Topic	Item** #**	Checklist item	Location where item is reported
TITLE			
Title	1	Identify the report as a systematic review.	Page 1
ABSTRACT			
Abstract	2	See the PRISMA 2020 for Abstracts checklist.	Page 2
INTRODUCTION			
Rationale	3	Describe the rationale for the review in the context of existing knowledge.	Page 3-9
Objectives	4	Provide an explicit statement of the objective(s) or question(s) the review addresses.	Page 10
METHODS			
Eligibility criteria	5	Specify the inclusion and exclusion criteria for the review and how studies were grouped for the syntheses.	Page 11
Information sources	6	Specify all databases, registers, websites, organisations, reference lists and other sources searched or consulted to identify studies. Specify the date when each source was last searched or consulted.	Page 10-11
Search strategy	7	Present the full search strategies for all databases, registers and websites, including any filters and limits used.	Page 10-11
Selection process	8	Specify the methods used to decide whether a study met the inclusion criteria of the review, including how many reviewers screened each record and each report retrieved, whether they worked independently, and if applicable, details of automation tools used in the process.	Page 12-13
Data collection process	9	Specify the methods used to collect data from reports, including how many reviewers collected data from each report, whether they worked independently, any processes for obtaining or confirming data from study investigators, and if applicable, details of automation tools used in the process.	Page 12-13
Data items	10a	List and define all outcomes for which data were sought. Specify whether all results that were compatible with each outcome domain in each study were sought (e.g. for all measures, time points, analyses), and if not, the methods used to decide which results to collect.	Page 12-13
	10b	List and define all other variables for which data were sought (e.g. participant and intervention characteristics, funding sources). Describe any assumptions made about any missing or unclear information.	Page 12-13
Study risk of bias assessment	11	Specify the methods used to assess risk of bias in the included studies, including details of the tool(s) used, how many reviewers assessed each study and whether they worked independently, and if applicable, details of automation tools used in the process.	Page 11-12
Effect measures	12	Specify for each outcome the effect measure(s) (e.g. risk ratio, mean difference) used in the synthesis or presentation of results.	Page 12
Synthesis methods	13a	Describe the processes used to decide which studies were eligible for each synthesis (e.g. tabulating the study intervention characteristics and comparing against the planned groups for each synthesis (item #5)).	Page 12
	13b	Describe any methods required to prepare the data for presentation or synthesis, such as handling of missing summary statistics, or data conversions.	N/A
	13c	Describe any methods used to tabulate or visually display results of individual studies and syntheses.	Page 14-17
	13d	Describe any methods used to synthesize results and provide a rationale for the choice(s). If meta-analysis was performed, describe the model(s), method(s) to identify the presence and extent of statistical heterogeneity, and software package(s) used.	Page 12-13
	13e	Describe any methods used to explore possible causes of heterogeneity among study results (e.g. subgroup analysis, meta-regression).	N/A
	13f	Describe any sensitivity analyses conducted to assess robustness of the synthesized results.	N/A
Reporting bias assessment	14	Describe any methods used to assess risk of bias due to missing results in a synthesis (arising from reporting biases).	N/A
Certainty assessment	15	Describe any methods used to assess certainty (or confidence) in the body of evidence for an outcome.	N/A
RESULTS			
Study selection	16a	Describe the results of the search and selection process, from the number of records identified in the search to the number of studies included in the review, ideally using a flow diagram.	Page 13-14
	16b	Cite studies that might appear to meet the inclusion criteria, but which were excluded, and explain why they were excluded.	N/A
Study characteristics	17	Cite each included study and present its characteristics.	Tables 1, 2
Risk of bias in studies	18	Present assessments of risk of bias for each included study.	N/A
Results of individual studies	19	For all outcomes, present, for each study: (a) summary statistics for each group (where appropriate) and (b) an effect estimate and its precision (e.g. confidence/credible interval), ideally using structured tables or plots.	Tables 1, 2
Results of syntheses	20a	For each synthesis, briefly summarise the characteristics and risk of bias among contributing studies.	Tables 1, 2
	20b	Present results of all statistical syntheses conducted. If meta-analysis was done, present for each the summary estimate and its precision (e.g. confidence/credible interval) and measures of statistical heterogeneity. If comparing groups, describe the direction of the effect.	Page 14-21
	20c	Present results of all investigations of possible causes of heterogeneity among study results.	N/A
	20d	Present results of all sensitivity analyses conducted to assess the robustness of the synthesized results.	N/A
Reporting biases	21	Present assessments of risk of bias due to missing results (arising from reporting biases) for each synthesis assessed.	N/A
Certainty of evidence	22	Present assessments of certainty (or confidence) in the body of evidence for each outcome assessed.	N/A
DISCUSSION			
Discussion	23a	Provide a general interpretation of the results in the context of other evidence.	Page 21-23
	23b	Discuss any limitations of the evidence included in the review.	Page 23-24
	23c	Discuss any limitations of the review processes used.	Page 23-24
	23d	Discuss implications of the results for practice, policy, and future research.	Page 21-22
OTHER INFORMATION			
Registration and protocol	24a	Provide registration information for the review, including register name and registration number, or state that the review was not registered.	Page 10
	24b	Indicate where the review protocol can be accessed, or state that a protocol was not prepared.	Page 10
	24c	Describe and explain any amendments to information provided at registration or in the protocol.	N/A
Support	25	Describe sources of financial or non-financial support for the review, and the role of the funders or sponsors in the review.	Page 25
Competing interests	26	Declare any competing interests of review authors.	Page 25
Availability of data, code and other materials	27	Report which of the following are publicly available and where they can be found: template data collection forms; data extracted from included studies; data used for all analyses; analytic code; any other materials used in the review.	Page 11

From: Page MJ, McKenzie JE, Bossuyt PM, Boutron I, Hoffmann TC, Mulrow CD, et al. The PRISMA 2020 statement: an updated guideline for reporting systematic reviews. BMJ 2021;372:n71. 10.1136/bmj.n71. This work is licensed under CC BY 4.0. To view a copy of this license, visit https://creativecommons.org/licenses/by/4.0/

## Appendix 2 Search strategy

Databases:

1. ERIC
2. PsycInfo
3. Scopus
4. Medline
5. Sociological Abstracts

**Searches executed:** 1/14/2025

## ProQuest

### #1

SU("Elementary School Students") OR SU("High School Students") OR SU("Junior High Students") OR SU(Teachers) OR SU("Elementary Schools") OR SU(“Middle Schools”) OR MAINSUBJECT.EXACT.EXPLODE("secondary schools") OR TI(school* or school-based or kindergarten* or student* or pupil* OR teacher*) OR AB(school* or school-based or kindergarten* or student* or pupil* or teacher*)

### #2

SU("Racial Differences") OR SU(racism) OR SU("Ethnicity") OR SU("Ethnic Groups") OR SU(“minority Groups”) OR SU(race) OR SU(“racial bias”) OR SU(“racial discrimination”) OR SU(“racial factors”) OR SU(“racial identification”) OR SU("Race") OR SU("Racial Differences") OR SU("Racial Identification") OR SU("Racial Composition") OR SU("Racial Relations") OR SU("Multiracial Persons") OR SU("African Americans") OR SU("White Students") OR SU("Minority Group Students") OR SU("African American Students") OR SU("Asian American Students") OR SU("Minority Groups") OR SU("Hispanic American Students") OR SU("American Indian Students") OR SU("Hispanic Americans") OR SU("Eskimos") OR SU("Spanish Americans") OR SU("Asians") OR SU("Blacks") OR SU("Asian Americans") OR SU("Ethnic Diversity") OR TI(race OR racial OR racist OR racism OR racially OR racialization OR microaggression* or micro-aggression* or bipoc or biracial OR bi-racial OR multiracial OR multi-racial OR anti-racis* Or antiracis* OR black OR “african american*” OR asian* OR white OR whites OR caucasian* OR hispanic* OR latina* OR latinx OR “native american*” OR “american indian*” OR indigenous OR eskimo* OR “spanish american*” OR discrim* OR bias* OR prejud* OR oppress* OR ethnic* OR cultur* OR religio* OR “minority group*” OR “minority student*” OR mistreat* OR “unfair treat*”) OR AB(race OR racial OR racist OR racism OR racially OR racialization OR microaggression* or micro-aggression* or multiracial OR bipoc or biracial OR bi-racial OR multi-racial OR anti-racis* Or antiracis* OR black OR “african american*” OR asian* OR white OR whites OR caucasian* OR hispanic* OR latina* OR latinx OR “native american*” OR “american indian*” OR indigenous OR eskimo* OR “spanish american*” OR discrim* OR bias* OR prejud* OR oppress* OR ethnic* OR cultur* OR religio* OR “minority group*” OR “minority student*” OR mistreat* OR “unfair treat*”)

### #3

MAINSUBJECT.EXACT.EXPLODE("Discipline") OR MAINSUBJECT.EXACT.EXPLODE("Discipline Policy") OR SU("discipline problems") OR TI((disciplin* N/6 (school* OR practice* OR referral* OR decision* OR problem* OR action*)) OR disciplinary OR punish* OR suspension* OR suspend* OR expulsion* OR expel* OR dismiss* OR “zero tolerance” OR “zero-tolerance” OR penal*) OR AB((disciplin* N/6 (school* OR practice* OR referral* OR decision* OR problem* OR action*)) OR disciplinary OR punish* OR suspension* OR suspend* OR expulsion* OR expel* OR dismiss* OR “zero tolerance” OR “zero-tolerance” OR penal*)

### #4

SU(intervention) OR SU("Positive Behavior Supports") OR SU("Professional Development") OR TI(intervention* OR policy OR policies OR framework* OR program* OR “professional development” OR training* OR action OR actions OR PBIS OR counsel* OR “positive behavior support*” OR strategy OR strategies) OR AB(intervention* OR policy OR policies OR framework* OR program* OR “professional development” OR training* OR action OR actions OR PBIS OR counsel* OR “positive behavior support*” OR strategy OR strategies)

### #5

(#1 AND #2 AND #3 AND #4)

## PsycInfo

### #1

(MH "Schools+") OR (MH "Students+") OR (MH "School Teachers") OR (TI school* OR AB school*) OR (TI school-based OR AB school-based) OR (TI student* OR AB student*) OR (TI kindergarten* OR AB kindergarten*) OR (TI pupil* OR AB pupil*) OR (TI teacher* OR AB teacher*)

### #2

(MH "racial bias") OR (MH “race and ethnic discrimination+”) OR (MH "prejudice") OR (MH "racism") OR (MH “antiracism”) OR (MH "racial and ethnic groups+") OR (MH “minority groups”) OR (TI race OR AB race) OR (TI racial OR AB racial) OR (TI racist OR AB racist) OR (TI racism OR AB racism) OR (TI racially OR AB racially) OR (TI racialization OR AB racialization) OR (TI microaggression* OR AB microaggression*) OR (TI micro-aggression* OR AB micro-aggression*) OR (TI biracial OR AB biracial) OR (TI bi-racial OR AB bi-racial) OR (TI bipoc OR AB bipoc) OR (TI multiracial OR AB multiracial) OR (TI multi-racial OR AB multi-racial) OR (TI anti-racis* OR AB anti-racis*) OR (TI antiracis* OR AB antiracis*) OR (TI black OR AB black) OR (TI "african american*" OR AB "african american*") OR (TI asian* OR AB asian*) OR (TI white OR AB white) OR (TI whites OR AB whites) OR (TI caucasian* OR AB caucasian*) OR (TI hispanic* OR AB hispanic*) OR (TI latina* OR AB latina*) OR (TI latinx OR AB latinx) OR (TI "native american*" OR AB "native american*") OR (TI "american indian*" OR AB "american indian*") OR (TI indigenous OR AB indigenous) OR (TI eskimo* OR AB eskimo*) OR (TI "spanish american*" OR AB "spanish american*") OR (TI discrim* OR AB discrim*) OR (TI bias* OR AB bias*) OR (TI prejud* OR AB prejud*) OR (TI oppress* OR AB oppress*) OR (TI ethnic* OR AB ethnic*) OR (TI cultur* OR AB cultur*) OR (TI religio* OR AB religio*) OR (TI "minority group*" OR AB "minority group*") OR (TI "minority student*" OR AB "minority student*") OR (TI mistreat* OR AB mistreat*) OR (TI "unfair treat*" OR AB "unfair treat*")

### #3

(MH "classroom discipline") OR (MH "school suspension") OR (MH "school expulsion") OR (TI (disciplin* N6 (school* OR practice* OR referral* OR decision* OR problem* OR action*))) OR (AB (disciplin* N6 (school* OR practice* OR referral* OR decision* OR problem* OR action*))) OR (TI punish* OR AB punish*) OR (TI suspension* OR AB suspension*) OR (TI suspend* OR AB suspend*) OR (TI expulsion* OR AB expulsion*) OR (TI expel* OR AB expel*) OR (TI dismiss* OR AB dismiss*) OR (TI "zero tolerance" OR AB "zero tolerance") OR (TI zero-tolerance OR AB zero-tolerance) OR (TI penal* OR AB penal*)

### #4

(MH “intervention”) OR (MH "school based intervention") OR (TI intervention* OR AB intervention*) OR (TI policy OR AB policy) OR (TI policies OR AB policies) OR (TI framework* OR AB framework*) OR (TI program* OR AB program*) OR (TI "professional development" OR AB "professional development") OR (TI training* OR AB training*) OR (TI action OR AB action) OR (TI actions OR AB actions) OR (TI PBIS OR AB PBIS) OR (TI counsel* OR AB counsel*) OR (TI "positive behavior support*" OR AB "positive behavior support*") OR (TI strategy OR AB strategy) OR (TI strategies OR AB strategies)

### #5

(#1 AND #2 AND #3 AND #4)

## Scopus

### #1

TITLE-ABS-KEY(School* or “school-based” or student* or kindergarten* or pupil* or teacher* )

### #2

Race OR "prejud*" OR "race factors" OR "minority group*" OR "population groups" OR "racial" OR "racist" OR "racism" OR "racially" OR "racialization" OR "microaggression*" OR "micro-aggression*" OR bipoc OR biracial OR bi-racial OR "multiracial" OR "multi-racial" OR "anti-racis*" OR "antiracis*" OR "black" OR “african-american” or "african american*" OR "asian*" OR "white" OR "whites" OR "caucasian*" OR "hispanic*" OR "latina*" OR "latinx" OR "native american*" OR "american indian*" OR "indigenous" OR "eskimo*" OR "spanish american*" OR "discrim*" OR "bias*" OR "oppress*" OR "ethnic*" OR "cultur*" OR "religio*" OR "minority student*" OR "mistreat*" OR "unfair treat*"

### #3

TITLE-ABS-KEY("punish*" OR (disciplin* W/6 (school* OR practice* OR referral* OR decision* OR problem* OR action*)) OR "suspension*" OR "suspend*" OR "expulsion*" OR "expel*" OR "dismiss*" OR "zero tolerance" OR "zero-tolerance" OR "penal*")

### #4

TITLE-ABS-KEY("intervention*" OR "policy" OR "policies" OR "framework*" OR "program*" OR "professional development" OR "training*" OR "action" OR "actions" OR "PBIS" OR "counsel*" OR "positive behavior support*" OR "strategy" OR "strategies")

### #5

(#1 AND #2 AND #3 AND #4)

## Medline

### #1

Exp Schools/ or exp Students/ or School Teachers/or (school* or school-based or student* or kindergarten* or pupil* or teacher*).tw,kw.

### #2

Exp race relations/ OR prejudice/ OR racism/ or race factors/ OR minority groups/ OR exp population groups or (race OR racial OR racist OR racism OR racially OR racialization OR microaggression* or micro-aggression* or bipoc OR biracial OR bi-racial OR multiracial OR multi-racial OR anti-racis* Or antiracis* OR black OR "african american*" OR asian* OR white OR whites OR caucasian* OR hispanic* OR latina* OR latinx OR "native american*" OR "american indian*" OR indigenous OR eskimo* OR "spanish american*" OR discrim* OR bias* OR prejud* OR oppress* OR ethnic* OR cultur* OR religio* OR "minority group*" OR "minority student*" OR mistreat* OR "unfair treat*").tw,kw.

### #3

Exp punishment/ OR ((disciplin* adj6 (school* OR practice* OR referral* OR decision* OR problem* OR action*)) OR punish* OR suspension* OR suspend* OR expulsion* OR expel* OR dismiss* OR "zero tolerance" OR "zero-tolerance" OR penal*).tw,kw.

### #4

Teacher training/ OR (intervention* OR policy OR policies OR framework* OR program* OR "professional development" OR training* OR action OR actions OR PBIS OR counsel* OR "positive behavior support*" OR strategy OR strategies).tw,kw.

### #5

(#1 AND #2 AND #3 AND #4)

## Sociological Abstracts

### #1

SU("Elementary Schools") OR SU("Secondary Schools") OR SU("Elementary School Students") OR SU("High School Students") OR SU("Junior High Students") OR SU(“Teachers”) or TI(school* or school-based or student* or kindergarten* or pupil* or teacher*) OR AB(school* or school-based or student* or kindergarten* or pupil* or teacher*)

### #2

SU(“discrimination”) OR SU(“racism”) OR SU(“racial differences”) OR SU(“race”) OR MAINSUBJECT.EXACT("Multiracial people") OR SU(“ethnic groups”) OR MAINSUBJECT.EXACT.EXPLODE(" Cultural Groups") OR TI(race OR "prejud*" OR "race factors" OR "minority group*" OR "population groups" OR "racial" OR "racist" OR "racism" OR "racially" OR "racialization" OR "microaggression*" OR "micro-aggression*" OR bipoc OR "multiracial" OR "multi-racial" OR "anti-racis*" OR "antiracis*" OR "black" OR “african-american” or "african american*" OR "asian*" OR "white" OR "whites" OR "caucasian*" OR "hispanic*" OR "latina*" OR "latinx" OR "native american*" OR "american indian*" OR "indigenous" OR "eskimo*" OR "spanish american*" OR "discrim*" OR "bias*" OR "oppress*" OR "ethnic*" OR "cultur*" OR "religio*" OR "minority student*" OR "mistreat*" OR "unfair treat*") OR AB(race OR "prejud*" OR "race factors" OR "minority group*" OR "population groups" OR "racial" OR "racist" OR "racism" OR "racially" OR "racialization" OR "microaggression*" OR "micro-aggression*" OR bipoc OR biracial OR bi-racial OR "multiracial" OR "multi-racial" OR "anti-racis*" OR "antiracis*" OR "black" OR “african-american” or "african american*" OR "asian*" OR "white" OR "whites" OR "caucasian*" OR "hispanic*" OR "latina*" OR "latinx" OR "native american*" OR "american indian*" OR "indigenous" OR "eskimo*" OR "spanish american*" OR "discrim*" OR "bias*" OR "oppress*" OR "ethnic*" OR "cultur*" OR "religio*" OR "minority student*" OR "mistreat*" OR "unfair treat*")

### #3

SU(“discipline”) OR SU(“punishment”) OR TI((disciplin* N/6 (school* OR practice* OR referral* OR decision* OR problem* OR action*)) OR disciplinary OR punish* OR suspension* OR suspend* OR expulsion* OR expel* OR dismiss* OR “zero tolerance” OR “zero-tolerance” OR penal*) OR AB((disciplin* N/6 (school* OR practice* OR referral* OR decision* OR problem* OR action*)) OR disciplinary OR punish* OR suspension* OR suspend* OR expulsion* OR expel* OR dismiss* OR “zero tolerance” OR “zero-tolerance” OR penal*)

### #4

SU(“intervention”) OR SU("training") OR SU("Professional training") OR TI(intervention* OR policy OR policies OR framework* OR program* OR “professional development” OR training* OR action OR actions OR PBIS OR counsel* OR “positive behavior support*” OR strategy OR strategies) OR AB(intervention* OR policy OR policies OR framework* OR program* OR “professional development” OR training* OR action OR actions OR PBIS OR counsel* OR “positive behavior support*” OR strategy OR strategies)

### #5

(#1 AND #2 AND #3 AND #4)
